# Epidemiology, Temporal Trends and Resistance Patterns of ESBL-Producing Non-Typhoidal *Salmonella* Isolated from Blood Cultures in Kisantu, DRC (2019–2022)

**DOI:** 10.3390/antibiotics15030271

**Published:** 2026-03-06

**Authors:** Jules Mbuyamba, Gaelle Nkoji-Tunda, Daniel Vita, Laurence Ngara, Edmonde Bonebe, Marie-France Phoba, Anne-Sophie Heroes, Mohamadou Siribie, Birkneh Tilahun Tadesse, Glody-Nickel Mbaa, Florian Marks, Liselotte Hardy, Jan Jacobs, Lisette Mbuyi-Kalonji, Octavie Lunguya

**Affiliations:** 1 Service of Microbiology, University Teaching Hospital of Kinshasa, Kinshasa P.O. Box 127, Democratic Republic of the Congo; ngaradisi@gmail.com (L.N.); mfphoba@yahoo.fr (M.-F.P.); lisettekalonji08@gmail.com (L.M.-K.);; 2 Department of Microbiology, National Institute for Biomedical Research, Kinshasa P.O. Box 1197, Democratic Republic of the Congo; gaellenkoji.gn@gmail.com (G.N.-T.); eddybonebe@yahoo.fr (E.B.); 3 Department of Basic and Clinical Sciences, Faculty of Medicine, Protestant University of Congo, Kinshasa P.O. Box 4745, Democratic Republic of the Congo; 4 Pediatric Infectious Diseases Department, Institute of Tropical Medicine (Nekken), Nagasaki University, Nagasaki 852-8523, Japan; 5 Ophthalmology Department, Saint Luc Hospital of Kisantu, Inkisi P.O. Box 221, Democratic Republic of the Congo; dvitamayimona@gmail.com; 6 Service of Medical Biology, Monkole Medical Center, Kinshasa P.O. Box 817, Democratic Republic of the Congo; 7 Department of Clinical Sciences, Institute of Tropical Medicine, 2000 Antwerp, Belgium; aheroes@itg.be (A.-S.H.);; 8 International Vaccine Institute, Seoul 07236, Republic of Korea; mohamadou.siribie@ivi.int (M.S.); birkneh.tadesse@ivi.int (B.T.T.);; 9 Burkina Institute of Global Health, Ouagadougou 10000, Burkina Faso; 10 Department of Global Health, Karolinska Institute, 141 86 Stockholm, Sweden; 11 Center for Innovative Drug Development and Therapeutic Trials for Africa, College of Health Sciences, Addis Ababa University, Addis Ababa 1000, Ethiopia; 12 Department of Internal Medicine, Ndjili Q7 General Hospital, Higher Institute of Medical Technology of Kinshasa, Kinshasa P.O. Box 190-200, Democratic Republic of the Congo; mglodinickel@gmail.com; 13 Service of Clinical Research, Saint Luc University Clinics, Catholic University of Louvain, 1200 Brussels, Belgium; 14 Madagascar Institute for Vaccine Research, University of Antananarivo, Antananarivo 101, Madagascar; 15 Department of Medicine, Cambridge Institute of Therapeutic Immunology and Infectious Disease, University of Cambridge, Cambridge CB2 0AH, UK; 16 Heidelberg Institute of Global Health, University of Heidelberg, 69120 Heidelberg, Germany; 17 The Hong Kong Jockey Club Global Health Institute, Hong Kong Special Administrative Region, China; 18 Department of Microbiology, Immunology and Transplantation, KU Leuven, 3000 Leuven, Belgium

**Keywords:** non-typhoidal *Salmonella*, ESBL, bloodstream infection, antimicrobial resistance, Kisantu, DRC

## Abstract

**Background:** Antimicrobial resistance (AMR), particularly due to extended-spectrum beta-lactamases (ESBL), is a growing threat to public health in sub-Saharan Africa. This study investigates the prevalence, epidemiological characteristics, resistance patterns and resistance dynamic over time of ESBL-producing non-typhoidal *Salmonella* (NTS) bacteremia in Kisantu, Democratic Republic of Congo (DRC), from 2019 to 2022. **Methods:** A retrospective observational study used routine bloodstream infection data from the AMR network at Saint Luc Hospital in Kisantu. Blood cultures from suspected bacteremia cases were processed using standard microbiological techniques. Bacterial identification relied on biochemical reactions. Antibiotic susceptibility testing and ESBL-producing NTS detection were performed by disk diffusion following Clinical and Laboratory Standards Institute guidelines. Associations between ESBL production and patient characteristics (age, sex) were assessed using Pearson’s Chi-square test, and annual temporal trends in ESBL-producing NTS from 2019 to 2022 were analyzed by logistic regression using 2019 as the reference year. **Results:** Of the 19,430 blood cultures, 1681 NTS isolates were identified, and 1568 of these were screened for ESBL. ESBL prevalence was significantly associated with age (*p* = 0.007), peaking in children under 2 years, but not with sex (*p* = 0.570). Compared with 2019, the likelihood of isolating ESBL-producing NTS increased markedly through 2022, with adjusted probabilities rising from 58% to 87%, reflecting a strong upward temporal trend. High levels of extensively drug-resistant (94.1%) were observed. No carbapenem resistance was detected. **Conclusions:** ESBL-producing NTS bacteremia is rising in Kisantu, DRC, mainly affecting children under 2 years. Rising resistance to key antibiotics limits treatment options and highlights the need for strengthened AMR surveillance, optimized antibiotic use, and vaccination strategies.

## 1. Introduction

Antimicrobial resistance (AMR) is a major global public health concern [1]. According to the World Health Organization (WHO), if no effective action is taken, AMR could become one of the leading causes of death by 2050, potentially causing up to 10 million deaths annually, with a particularly severe impact in sub-Saharan Africa [2].

One of the main components of AMR is bacterial resistance to antibiotics, which was observed very soon after the discovery of the first antibiotic thanks to the work of Alexander Fleming in 1928 [3,4]. This phenomenon has rapidly spread throughout the world with the emergence of resistance to newly introduced antibiotics. As an example, following the introduction of third-generation cephalosporins in the late 1970s, the first *Klebsiella pneumonia* isolates producing extended-spectrum beta-lactamases (ESBL) were detected in Germany [3,5,6]. ESBL constitute a heterogeneous group of enzymes capable of hydrolyzing a wide range of beta-lactam antibiotics, including penicillins, first- and second-generation cephalosporins, as well as third- and fourth-generation cephalosporins and monobactams [6,7]. This broad enzymatic activity substantially complicates the therapeutic management of infected patients, leading to prolonged hospitalization and death [2].

Today, ESBL-producing bacteria are increasingly common, particularly among Enterobacterales such as *Escherichia coli*, *Klebsiella pneumoniae*, and non-typhoidal *Salmonella* (NTS) [6,7,8,9]. NTS are bacteria capable of colonizing both humans and animals and are primarily transmitted via the fecal–oral route, mainly through the consumption of contaminated food [9]. Although they are most commonly associated with diarrheal disease worldwide, NTS represent one of the leading causes of invasive infections in sub-Saharan Africa, specifically in immunocompromised adults and children with severe malaria, anemia and malnutrition [9,10,11,12].

In 2017, Stanaway et al. estimated that 535,000 (95% uncertainty interval 409,000–705,000) cases of invasive NTS occurred globally, with the highest incidence observed in sub-Saharan Africa, especially among children younger than 5 years old [34.3 (23.2–54.7) cases per 100,000 person-years] [13]. Bloodstream infections constitute the main manifestation of invasive NTS infections. Although also reported on other continents, cases of NTS bacteremia remain for more than 50% the prerogative of sub-Saharan African countries [11,14,15,16,17,18,19]

In the Democratic Republic of Congo (DRC), a bloodstream infection and antimicrobial resistance surveillance network that has been active for several years reported NTS as the leading cause of bacteremia, with an estimated mortality rate of 22% among children under 5 years old [12,17,18,20,21]. Further, several studies conducted over time in the DRC have documented changes in the antimicrobial resistance profiles of NTS isolates, notably showing an increase in the proportion of ESBL-producing NTS isolates from nearly 1.1% in 2012 to approximately 12.7% of *Salmonella* Typhimurium isolates in 2015 [18,20]. At the Kisantu site, located in the southwestern part of the DRC, the proportion of NTS isolates resistant to ceftriaxone increased from 0% in 2012 to nearly 15% in 2020 [12,21]. This situation is of high concern, as over 95% of empirical treatments for suspected bacteremia at Saint Luc Hospital (Kisantu Health Zone, one of the surveillance sites in DRC) rely on third-generation cephalosporins [22].

The present study aims to describe the prevalence, epidemiological characteristics, resistance profiles, and temporal dynamics of ESBL-producing NTS isolated from blood cultures collected at Saint Luc Hospital. This work addresses an existing research gap, as recent data on ESBL-producing NTS remain scarce, with most available studies dating back several years.

## 2. Results

### 2.1. Blood Culture Outcomes and NTS Isolation

**Figure 1:**
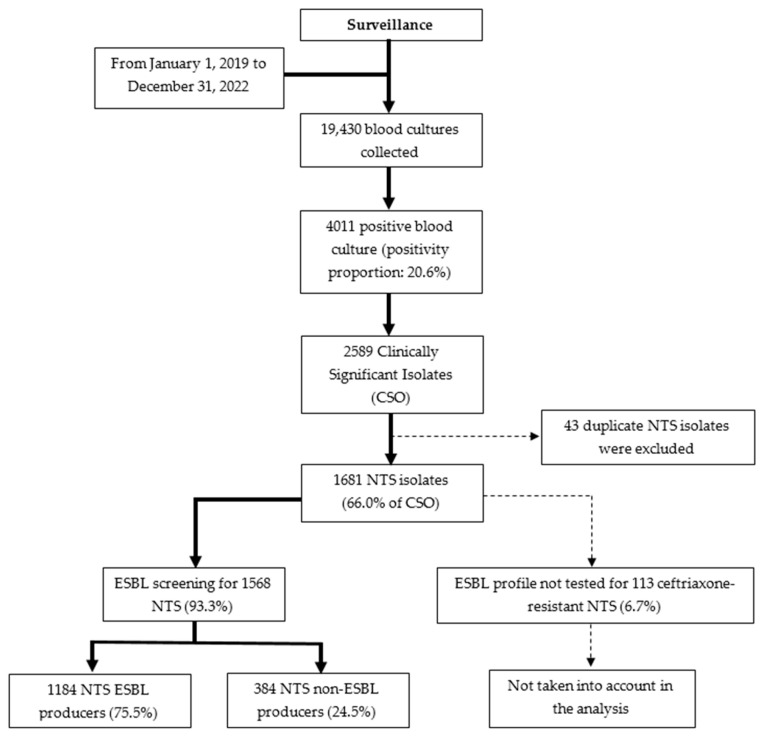
Breakdown of blood culture results obtained at Saint Luc Hospital. Solid arrows (→) indicate normal progression of cases, while dashed arrows (---) represent excluded cases.

Between January 2019 and December 2022, a total of 19,430 blood cultures were collected at Saint Luc Hospital. NTS represented the majority of clinically significant isolates, with a substantial proportion confirmed as ESBL-producing NTS isolates (75.5%) (Figure 1).

### 2.2. Epidemiologic Characteristics of Patients with ESBL-Producing NTS Bacteremia

Overall, the majority of NTS isolates was observed among children under 2 years old (78.0%), with a significant difference across age groups (*p* = 0.007) (Table 1). There was no significant difference in the proportion of ESBL-producing NTS between males and females (*p* = 0.570).

### 2.3. Temporal Trends of ESBL-Producing NTS Isolates from 2019 to 2022

The odd ratio of isolating ESBL-producing NTS isolates increased significantly in 2020 (OR = 2.63), 2021 (OR = 2.75) and 2022 (OR = 4.72), with the highest increase observed in 2022 (Table 2). The aggregated detailed data on proportions of ESBL-producing NTS are displayed by quarter in Supplementary Figure S1.

**Figure 2:**
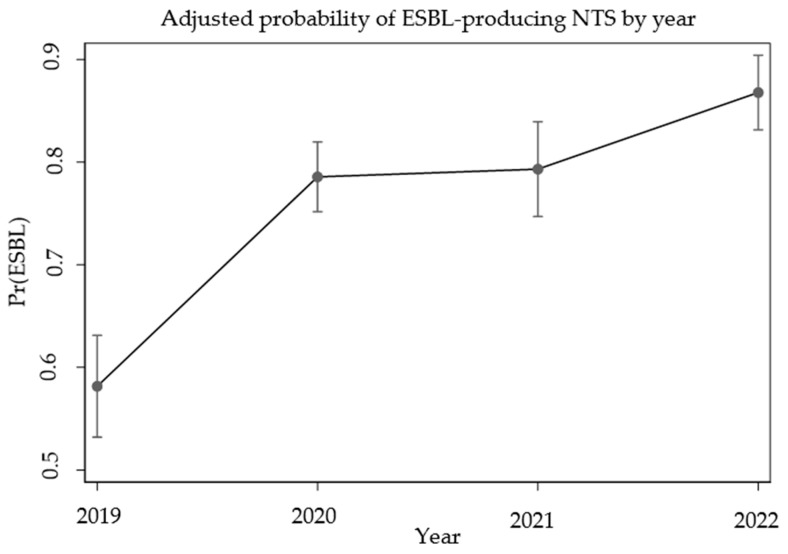
Adjusted probability of ESBL-producing NTS isolates from 2019 to 2022.

Simple logistic regression showed that the adjusted probabilities of isolating ESBL-producing NTS increased significantly between 2019 and 2022, rising from 58% (95% CI: 53–63%) to 87% (95% CI: 84–90%), suggesting an increasing trend in the number of cases over the years (Figure 2).

### 2.4. Resistance Profile of ESBL-Producing NTS Isolates

Among ESBL-producing NTS isolates, resistance to conventional antibiotics was high, whereas susceptibility to carbapenems was retained (Table 3). The majority of isolates exhibited an Extensively Drug-Resistant (XDR) profile (94.1%).

It should be noted that azithromycin susceptibility testing was performed only between September 2021 and December 2022 for 345 isolates, among which 6.4% exhibited resistance; these data are not included in the main table. Only 6.3% of ESBL-producing NTS isolates were Pan-Drug-Resistant (PDR).

## 3. Discussion

In terms of clinically significant bacteria, NTS is the leading cause of bloodstream infections at Saint Luc Hospital in Kisantu. This is consistent with findings from previous surveillance studies in Kisantu, DRC and other regions of sub-Saharan Africa [9,11,12,18,20,23].

The 75.5% proportion of ESBL production among NTS isolates in this study marks a sharp increase compared to historical data from DRC: 0.0% in 2010–2011 (Phoba et al.) and less than 2% between 2007–2021 (Lunguya et al.) [20,21]. This trend highlights the alarming progression of resistance, likely driven by antibiotic overuse or misuse, as well as by changes associated with evolving serotypes [12,22]. However, due to limited access to laboratory supplies on the local market, particularly antimicrobial susceptibility testing disks, the ESBL profile could not be determined for 113 ceftriaxone-resistant NTS isolates (6.7%), which would likely have increased the proportion of ESBL-producing NTS isolates (Figure 1).

Most ESBL-producing NTS isolates were recovered from children under 2 years of age, confirming their greater vulnerability to invasive infections. This increased susceptibility may be explained by the immaturity of the immune system in children younger than 2 years. Beyond immune system immaturity, children in the study region are also exposed to other risk factors, including malaria, malnutrition, and anemia, which increase their likelihood of developing the disease and contribute to the overuse of antibiotics. Their overexposure may also help explain the surge in antimicrobial resistance [22,24,25]. In the present study, age was significantly associated with ESBL-producing NTS infections (*p* = 0.007), whereas no significant association was observed with sex (*p* = 0.570). This finding is consistent with the usual pattern of invasive non-typhoidal *Salmonella* infections, which predominantly affect young children, and supports previous observations reported by several authors, including Tack et al. and Kalonji et al., in DRC [12,18].

The analysis of annual data revealed a significant upward trend in ESBL-producing NTS prevalence over the study period. This trend may be influenced by various unmeasured factors, such as antibiotic selective pressure, infection control practices, or environmental conditions; however, their specific impact was not assessed in the present study.

Extensively drug-resistant (XDR) isolates were detected at very high proportions in the present study, in contrast to the low proportions reported by Tack et al. for NTS isolates from Kisantu between 2015–2017 [12]. As described by other authors, the high proportions of XDR strains could be explained by the co-occurrence of plasmid-borne resistance genes, with ESBL genes often associated with other genes that confer resistance to multiple antibiotic families [7]. The level of resistance to ciprofloxacin was significantly elevated in comparison to previously documented statistics from the DRC and other African regions [11,12,18,20,21]. This sharp increase may reflect a growing selective pressure due to the widespread use of fluoroquinolones and raises concern about the future efficacy of this class of antibiotics.

Our results revealed no resistance to carbapenems. Access to these antibiotics remains limited for the population of Kisantu due to their high cost, consistent with observations from other studies conducted in certain African countries [26].

Given the high resistance profile of NTS isolates to commonly used antibiotics and limited patient access to meropenem, the management of ESBL-producing NTS infections often necessitates the use of azithromycin, which continues to show a low resistance proportion. Due to its accessibility, azithromycin has become one of the most widely used antibiotics at Saint Luc Hospital of Kisantu. Nevertheless, its use is supported by only a few clinical guidelines, primarily as an oral treatment during the non-acute phase of the disease [27,28]. A prospective observational study conducted in Kisantu by Tack (2021–2022) showed that azithromycin, used according to susceptibility profiles, improves survival in children under 5 years hospitalized with NTS bloodstream infections [29].

The emergence of XDR NTS isolates in this study highlights a critical gap between current laboratory antibiotic susceptibility testing practices and clinical needs in high-resistance contexts while also emphasizing the limitations of currently recommended antibiotic panels [30]. These limitations restrict clinicians’ ability to identify alternative therapeutic options and increase the risk of overusing the few remaining active antibiotics, which potentially accelerates the spread of antibiotic resistance. 

This context calls for moving beyond the exclusive surveillance of guideline-listed antibiotics toward a proactive evaluation of alternative agents. Newer β-lactam/β-lactamase inhibitor combinations, such as ceftazidime-avibactam, meropenem-vaborbactam and ceftolozane-tazobactam, as well as cefiderocol, represent short-term options for studies aimed at expanding the antibiotic panel for testing invasive XDR NTS isolates. Although these combinations and cefiderocol are primarily recommended by the Infectious Diseases Society of America for carbapenem-resistant organisms (IDSA 2024), they display excellent activity against ESBL-producing Enterobacterales, making them strong candidates for research in this context [31].

Generating MIC distribution data for these alternative antibiotics against ESBL-producing NTS is an urgent next step for research in order to proactively identify and anticipate alternative salvage therapeutic options when standard treatments fail.

These results also highlight the importance of regularly updating clinical treatment guidelines based on laboratory-derived antibiotic resistance surveillance data in order to optimize patient management.

The danger posed by increasing antibiotic resistance among ESBL-producing NTS lies in the fact that these bacteria are capable of circulating between humans, animals and the environment. Consequently, the spread of ESBL-producing NTS represents a major public health concern, highlighting the urgent need to curb the development and spread of antibiotic resistance. The Democratic Republic of the Congo (DRC), one of the poorest countries worldwide, is unfortunately facing several factors that may explain the surge in antibiotic resistance, notably the inappropriate and excessive use of antibiotics and insufficient hygiene measures [22,25]. Without effective interventions to contain antibiotic resistance, the situation currently observed in Kisantu could continue to spread in the coming years not only in the DRC but also to other regions or countries.

### Strengths and Limitations

Blood cultures were embedded in routine patient care and a national surveillance project, ensuring consistent and uninterrupted sampling over time. This study represents the first investigation in the DRC to present the temporal trends of ESBL-producing NTS isolates. All isolates collected as part of the surveillance have been preserved, providing a valuable resource for future complementary analyses. These isolates will be used to contribute as soon as possible to the future extension of the antibiotic testing panel, providing a key resource for future breakpoint establishment, as was successfully achieved previously for azithromycin in invasive NTS infections [32]. It is desirable, in every case, to systematically validate the new antibiotic panel through laboratory analyses and assessments of its clinical effectiveness.

The present study has some limitations. A major limitation is that the antibiotic panel tested for the ESBL-producing NTS did not take into account certain alternative antibiotic agents, which limits the immediate clinical applicability of our findings.

Secondly, the molecular characterization of the isolates, including the confirmation of the ESBL genes involved, has not yet been performed and remains a step for future investigation. Further, azithromycin resistance was tested for only a small subset of NTS isolates, and Minimal Inhibitory Concentration (MIC) values were not determined; however, a previous study demonstrated an excellent correlation between disk diffusion results and MIC values [32]. Additionally, serotype determination was not performed; nevertheless, *Salmonella* Typhi was reliably distinguished from NTS using biochemical reactions. In the context of this temporal dynamics analysis, the potential impact of confounding factors has not yet been assessed and will need to be evaluated in future works.

## 4. Materials and Methods

### 4.1. Study Design

A retrospective descriptive observational study with an analytical aim was conducted at Saint Luc Hospital in Kisantu, DRC, from January 2019 to December 2022. This investigation is part of the national AMR surveillance Network established by the National Institute for Biomedical Research (INRB) in collaboration with the Institute of Tropical Medicine Antwerp (ITM) [12,18,20]. The surveillance system, initiated in 2007, has been running continuously and involves the systematic collection of blood cultures from all patients presenting with suspected bacteremia.

### 4.2. Study Population

For the purpose of this study, the inclusion criterion was all patients with a positive blood culture for NTS. Analyses excluded patients with bacteremia due to any other bacteria, e.g., *Salmonella* Typhi. Deduplication was performed at the isolate level to ensure that only one isolate per episode of bloodstream infection per patient was included. An episode was defined as any blood culture collected within a period of ≤14 days from the same patient.

In addition, NTS isolates for which the ESBL profile had not been investigated by the laboratory were excluded, despite showing resistance to ceftriaxone. Some of the data were collected during the implementation of the Severe Typhoid in Africa (SETA) study in Kisantu (2017–2020), and additional data were collected at the beginning of the implementation of the Effect of a Novel Typhoid Conjugate Vaccine in Africa (THECA) study (2021–2022) [33,34].

### 4.3. Study Site

Saint Luc Hospital is the only general referral hospital in the Kisantu Health Zone, located in the Province of Kongo Central (southwestern part of the DRC). The health zone of Kisantu covers an area of about 2400 km^2^, with a total population of approximately 200,000 inhabitants. The health zone is currently subdivided into 18 health areas, each comprising a health center responsible for patient care and referral to the hospital in accordance with national guidelines [12].

Since 2007, the hospital has been part of the national surveillance network for bacterial antibiotic resistance with the integration of blood cultures into routine clinical practice [12,20,35]. *Plasmodium falciparum* malaria, one of the main risk factors for the occurrence of NTS invasive infections, remains endemic in Kisantu Health Zone, as in the rest of the country [24,36].

### 4.4. Sample Collection

Blood cultures were collected from patients presenting at Saint Luc Hospital of Kisantu with clinical suspicion of bloodstream infections, including, among others, fever, suspected neonatal infection and severe localized infections (such as pneumonia, meningitis, complicated urinary tract infections) with potential progression to bacteremia. Collection procedures followed standardized protocols, including those described by Tack et al. [12]. After collection in the various hospital departments, the blood cultures, along with the accompanying analysis request forms, were immediately transported to the laboratory at room temperature for analysis.

### 4.5. Laboratory Analysis

Upon reception in the laboratory, blood culture bottles (BacT/ALERT, Marcy-L’Etoile, France) were incubated at 37 °C for up to seven days. Daily monitoring was performed to detect color changes at the base sensor of the bottles, indicating microbial growth. Any positive bottle was immediately removed from the incubator and processed for further analysis. After removing the positive bottle from the incubator, a microscopic examination, including a wet mount and Gram staining, was performed, and the preliminary result was immediately communicated to the clinician. On the same day, the positive blood culture broth was subcultured onto blood agar and MacConkey agar to isolate the causative bacteria. The identification of NTS isolates was performed using a standard biochemical panel (Table 4) [20] based on two specific biochemical characteristics that allowed us to exclude *Salmonella* Typhi: namely, the ability to metabolize citrate on Simmons citrate medium and positive ornithine decarboxylase (ODC) activity.

**Figure 3:**
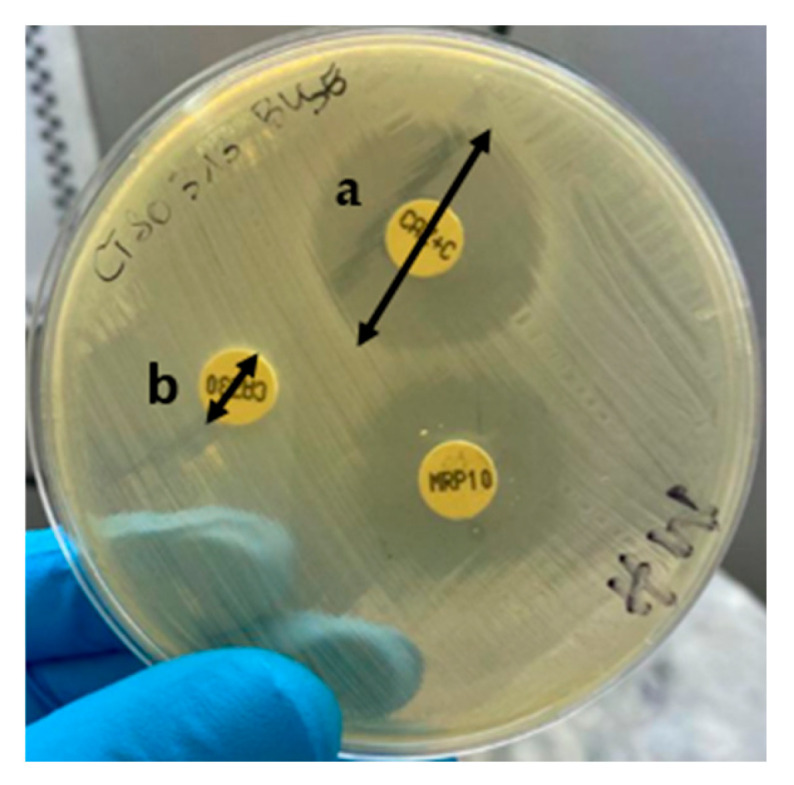
Illustrative image of an ESBL-producing NTS isolate. **a** = diameter around ceftazidime disk combined with clavulanic acid; **b** = diameter around ceftazidime disk without clavulanic acid.

On Mueller–Hinton agar, antibiotic susceptibility testing was performed by the Kirby Bauer disk diffusion method using Clinical and Laboratory Standards Institute (CLSI) guidelines, updated annually from 2019 to 2022 [36,37,38,39]. The interpretation of the antibiotic susceptibility test results was based on the breakpoint diameters provided by the Clinical and Laboratory Standards Institute (CLSI) guidelines, updated annually from 2019 to 2022. ESBL production was detected using the combined disk method, a ≥ 5 mm increase in the diameter of the inhibition zone around a cefotaxime or ceftazidime disk when combined with clavulanic acid, compared to the zone produced by the same disk(s) without clavulanic acid (Figure 3) [37,40].

In addition, the antibiotic panel tested included ampicillin (10 µg), ceftriaxone (30 µg)/cefotaxime (30 µg), trimethoprim-sulfamethoxazole (1.25/23.75 µg), chloramphenicol (30 µg), pefloxacin (5 µg), azithromycin (15 µg) and imipenem (10 µg)/meropenem (10 µg). NTS isolates were characterized as ciprofloxacin-resistant when they showed resistance to the pefloxacin disk, according to the breakpoint diameters provided by the Clinical and Laboratory Standards Institute (CLSI) guidelines, updated annually from 2019 to 2022 [37,38,39,40].

The NTS were characterized XDR (Extensively Drug-Resistant) when exhibiting co-resistance to ampicillin, trimethoprim-sulfamethoxazole, chloramphenicol, ceftriaxone and pefloxacin and were characterized PDR (Pan-Drug-Resistance) when exhibiting co-resistance to ampicillin, trimethoprim-sulfamethoxazole, chloramphenicol, ceftriaxone, pefloxacin, and azithromycin [12].

Concerning azithromycin, it was introduced in the laboratory in September 2021, following the surge in resistance among NTS and the promising susceptibility results reported by Tack et al. [32]. In the present study, for the interpretation of azithromycin-susceptibility testing results, we applied the clinical breakpoints defined by CLSI for *Salmonella* Typhi in 2021 and 2022 [37,38,39,40], as recommended given the absence of validated breakpoints for NTS. This approach is supported by Tack et al., who highlighted the relevance of the *Salmonella* Typhi ECOFF for guiding azithromycin-susceptibility interpretation in NTS isolates [32]. Ultimately, azithromycin susceptibility was assessed for only 345 NTS isolates out of the total collection of ESBL-producing NTS.

It should be noted that, at the conclusion of each analysis, a final result was communicated to the clinician for patient management, and the identified strains were preserved for the purposes of national AMR surveillance.

### 4.6. Data Collection and Statistical Analysis

Data were recorded using Microsoft Excel 2016 (Redmond, WA, USA) and analyzed using Stata 18 (StataCorp, College Station, TX, USA).

The dependent variable was the production of extended-spectrum β-lactamases by NTS isolates. Independent variables included the date of blood culture collection, patient age, sex, and the resistance profile of NTS to various antibiotics. Descriptive statistics were presented using frequency tables and figures. Only the first isolate per bloodstream infection episode was considered for analysis, and control blood culture cases were also excluded.

Participants were stratified into four age groups (<2 years, 2–4 years, 5–14 years, and ≥15 years) to reflect relevant biological and epidemiological differences in susceptibility to NTS infections. Pearson’s Chi-square test was used to assess the association between extended-spectrum β-lactamase (ESBL) production by NTS (dependent variable) and explanatory variables, including patient sex and age. The temporal trend of ESBL-producing NTS cases from 2019 to 2022 was analyzed using simple logistic regression. The dependent variable was the presence of ESBL, coded as a dichotomous variable (1 = Yes, 0 = No), while the year of NTS isolation was used as the explanatory variable, with 2019 serving as the reference year.

A significance threshold of *p* < 0.05 was applied.

Resistance proportions for each antibiotic were calculated among ESBL-producing isolates.

## 5. Conclusions

This study highlights a critical and escalating public health concern in Kisantu, DRC, due to the high prevalence of ESBL-producing NTS bacteremia between 2019 and 2022. More than three-quarters of all NTS isolates were ESBL producers. While the proportions of ESBL-producing NTS isolates were relatively similar across all age groups, children under 2 years carried the highest absolute burden. Similar proportions between males and females indicate the widespread dissemination of resistant isolates throughout the population.

The temporal analysis demonstrated a statistically significant increase in ESBL-producing NTS prevalence, suggesting a growing selective pressure likely driven by the widespread empirical use of third-generation cephalosporins. Additionally, the high proportions of XDR further restrict available therapeutic options and raise urgent concerns regarding the effectiveness of first-line antibiotics. If this upward trend in bacterial resistance continues over the coming years and no effective measures are implemented to curb antimicrobial resistance, it may seriously compromise patient management and adversely affect treatment outcomes and patient recovery.

These findings underscore the urgent need to strengthen antimicrobial resistance surveillance, promote rational antibiotic prescribing policies, and invest in molecular studies to better understand resistance mechanisms. Furthermore, vaccination, combined with targeted interventions, remains essential to protect vulnerable pediatric populations and preserve the effectiveness of available treatment options.

## Figures and Tables

**Table 1:** Age and gender distribution of ESBL-producing NTS isolates.

Characteristics	NTS Isolates		*p*-Value
	ESBL-producing NTS *n* (%)	Non-ESBL-producing NTS *n* (%)	
**Age groups**			0.007
<2 years	853 (78.0)	241 (22.0)	
2–4 years	245 (70.0)	105 (30.0)	
5–14 years	59 (67.8)	28 (32.2)	
≥15 years	27 (73.0)	10 (27.0)	
**Sex**			0.570
Male	641 (76.4)	198 (23.6)	
Female	543 (74.5)	186 (25.5)	

**Table 2:** Simple logistic regression analysis of the temporal trends of ESBL-producing NTS isolates.

Year	Odd Ratio (OR)	95% CI	*p*-Value
2019	Reference	-	-
2020	2.6	2.0–3.5	<0.001
2021	2.8	1.9–3.9	<0.001
2022	4.7	3.2–6.9	<0.001

CI: 95% confidence interval.

**Table 3:** Resistance profile of ESBL-producing NTS to antibiotics.

Antibiotics/Resistance Mechanism	Resistance *n* (%)
Ampicillin	1184 (100.0)
Ceftriaxone/cefotaxime	1184 (100.0)
Trimethoprim-sulfamethoxazole	1169 (98.8)
Chloramphenicol	1174 (99.2)
Ciprofloxacin	117 (94.3)
Imipenem/meropenem	0 (0.0)
XDR	1114 (94.1)

**Table 4:** Biochemical tests employed for the identification of NTS.

	UREASE	GLUCOSE	LACTOSE	GAS	H_2_S ^1^	MOBILITY	CITRATE	INDOLE	LDC ^2^	ONPG ^3^	ODC ^4^
Non-typhoidal *Salmonella*	-	+	-	+	+	+	+	-	+	-	+

^1^ Hydrogen sulfide; ^2^ Lysine decarboxylase; ^3^ Para Nitrophenyl-Galacto Pyranoside; ^4^ Ornithine Decarboxylase. ’’+’’ indicates a positive biochemical reaction; ’’-‘’ indicates a negative biochemical reaction.

## Data Availability

The data used in this study come from an extract of the database of the National Multicenter Surveillance Network for Bacteremia and Antimicrobial Resistance, specifically corresponding to the Kisantu site. The full database is held by the National Institute of Biomedical Research (INRB) in the Department of Bacteriology and Research. The datasets analyzed are not publicly available due to confidentiality and ethical restrictions but can be obtained from the corresponding author upon reasonable request and with authorization from the surveillance network coordinators.

## Supplementary Materials

The following supporting information can be downloaded at: https://www.mdpi.com/article/10.3390/antibiotics15030271/s1, Figure S1: Quarterly (Q) distribution of ESBL-producing NTS isolates from 2019 to 2022.

## Abbreviations

The following abbreviations are used in this manuscript:

AMR	Antimicrobial resistance
CI	Confidence interval
CLSI	Clinical and Laboratory Standards Institute
CSO	Clinically significant isolate
DRC	Democratic Republic of the Congo
ECOFF	Epidemiological cut-off
ESBL	Extended-spectrum beta-lactamase
H_2_S	Hydrogen sulfide
IDSA	Infectious Diseases Society of America
INRB	Institut National de Recherche Biomédicale
ITM	Institute of Tropical Medicine Antwerp
LDC	Lysine decarboxylase
MIC	Minimal inhibitory concentration
NTS	Non-typhoidal *Salmonella*
ODC	Ornithine decarboxylase
ONPG	Para nitrophenyl-galacto pyranoside
PDR	Pan-drug-resistance/resistant
Pr	Probability
Q	Quarter
SETA	Severe Typhoid in Africa
THECA	The Effect of a Novel Typhoid Conjugate Vaccine in Africa
WHO	World Health Organization
XDR	Extensively drug-resistance/resistant
