# Supplementary material for: Epidemiology, Temporal Trends and Resistance Patterns of ESBL-Producing Non-Typhoidal Salmonella Isolated from Blood Cultures in Kisantu, DRC (2019–2022)
Source: Antibiotics (Basel). 2026 Mar 6;15(3):271. doi: 10.3390/antibiotics15030271 (PMC13023545; doi:10.3390/antibiotics15030271)
Supplement: Supplementary file 1 [file antibiotics-15-00271-s001.zip › antibiotics-4020067-supplementary.pdf]

SUPPLEMENTARY INFORMATION

Figure S1. Quarterly (Q) distribution of ESBL-producing NTS isolates from 2019 to 2022

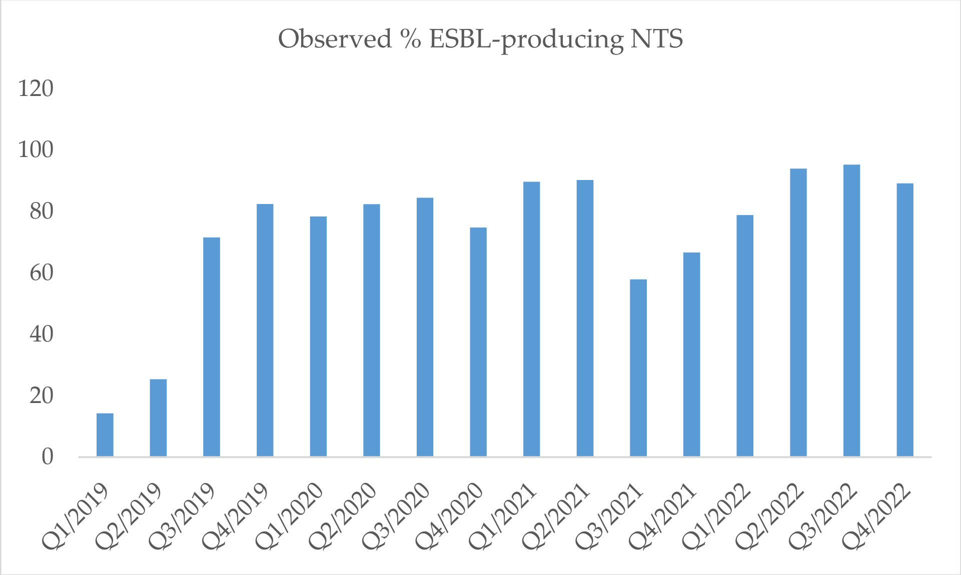

A clear increasing trend in ESBL prevalence was observed, from 14.3% in Q1/2019 to 95.3% to Q3/2022.
